# Peripapillary Vessel Density in Young Patients with Open-Angle Glaucoma: Comparison between High-Tension and Normal-Tension Glaucoma

**DOI:** 10.1038/s41598-019-55707-5

**Published:** 2019-12-16

**Authors:** Ji-Hye Park, Chungkwon Yoo, Yong Yeon Kim

**Affiliations:** Department of Ophthalmology, Korea University College of Medicine, Seoul, Korea

**Keywords:** Glaucoma, Optic nerve diseases

## Abstract

Although primary open-angle glaucoma (OAG) generally occurs in older individuals and manifests in eyes with elevated intraocular pressure (IOP), it may also occur in young patients or in eyes with an IOP that always measures within the statistically normal range. Recent advances in optical coherence tomography angiography have enabled noninvasive visualization of the vasculature around the optic disc. In this study, we investigated the clinical features of young Korean patients with OAG and compared the peripapillary vessel density of patients with normal-tension glaucoma (NTG) to those with high-tension glaucoma (HTG). The peripapillary vessel density was reduced in eyes with HTG compared with that in normal subjects (HTG: 23.18 ± 2.06% vs. normal subjects: 24.74 ± 1.88%, *P* value = 0.013). In contrast, the peripapillary vessel density of eyes with NTG was comparable with that of normal eyes (NTG: 23.98 ± 2.30% vs. normal subjects: 24.74 ± 1.88%, *P* value = 0.505). These findings suggest that young patients with HTG show greater peripapillary microvascular attenuation than healthy subjects or young patients with NTG, indicating that different levels of the initial untreated IOP may have different effects on the peripapillary vessel density in young patients with OAG.

## Introduction

Glaucoma is a progressive optic neuropathy, resulting from apoptosis of the retinal ganglion cells. It shows a characteristic structural loss and corresponding visual field (VF) defects and is a leading cause of irreversible blindness worldwide^1,2^. It usually occurs in eyes with high intraocular pressure (IOP) and in older subjects^1^. However, glaucomatous optic neuropathy may develop in young patients or in eyes with an IOP that measures within the statistically normal range^3^. Juvenile-onset glaucoma (JOAG) has been associated with autosomal-dominant inheritance, and the affected eyes are often myopic. Compared with adult-onset open-angle glaucoma (OAG), the response to IOP-lowering medication or laser treatment is generally poor and surgical intervention is often required in patients with JOAG^4^. In addition, the success rate of filtering surgery is lower in JOAG patients than that in adult-onset OAG patients, which can lead to early visual impairment^5^. On the other hand, previous reports demonstrated that glaucomatous eyes with IOP within the statistically normal range (normal-tension glaucoma, NTG) progress at a relatively slower rate than eyes with elevated IOP (high-tension glaucoma, HTG)^6^. Although NTG has often been considered as part of the primary OAG spectrum, several reports indicate distinct features of structural or functional damage that differ from eyes with HTG^7–9^. Moreover, significant evidence suggests that primary vascular factors, such as migraine headache, Raynaud’s phenomenon, disc hemorrhage, and orthostatic hypotension, may play a role in the development and progression of glaucomatous damage in eyes with NTG^10–12^. Thus, non-IOP factors may play a role in the pathogenesis of NTG.

Previously, two population-based studies of Asians demonstrated that narrowing of the retinal arterioles was associated with glaucomatous optic neuropathy^13,14^. In a study investigating the retinal vessel diameter in young patients with OAG, Lee and colleagues reported that the mean central retinal arteriolar equivalent was smaller in the NTG group than in the HTG group, while the mean central retinal venular equivalent showed no intergroup differences^15^. Recently, Adiarti and colleagues conducted a comparative evaluation of the retinal vessel diameter in young adults with glaucomatous optic disc and normal IOP and age- and sex-matched normal subjects^16^. The retinal arteriolar narrowing was associated with glaucomatous optic disc in young adults, independent of an increase in IOP. These studies suggested that the reduced ocular blood flow from a narrow central retinal arteriole in young patients with NTG might induce glaucomatous damage, which supports the vascular theory of the pathogenesis of glaucomatous optic neuropathy. However, it remains unclear whether such association is a cause or a result of glaucomatous damage.

Optical coherence tomography angiography (OCTA) provides quantitative and qualitative information about the microvasculature of the retinal and choroidal layers^17,18^. Several studies using OCTA demonstrated that the vascularity in the peripapillary area was impaired in patients with glaucoma and there was a significant association between the severity of glaucoma and the amount of peripapillary vessel density reduction^18–22^. In addition to the decreased peripapillary vessel density, choroidal microvascular dropout (MvD) was also noted in patients with glaucoma^23^. Some researchers have associated their OCTA findings with the vascular aspect of the NTG pathogenesis^24,25^. However, these studies included older subjects with possibly co-existing systemic diseases that could affect the ocular blood flow and/or physiologic aging^10,17^. Few studies have reported a quantitative analysis of the peripapillary microvasculature in eyes of young patients with OAG. In this study, we evaluated the vessel density around the optic disc using OCTA in young patients with OAG, and compared the peripapillary vessel density and structural features of optic disc between NTG and HTG groups.

## Results

In the present study, we reviewed the medical records of 201 young patients with OAG, excluding 97 because of the following reasons: previous history of ocular or refractive surgery (37 patients), preperimetric glaucoma (23 patients), secondary glaucoma (18 patients), refractive error <−9 diopters (7 patients), usage of steroid eyedrops (7 patients), and history of medical diseases (5 patients). One-hundred and four young patients with OAG and a single eye from each of 30 normal subjects were enrolled. The patients’ demographics are listed in Supplementary Table. To compare the features of the three groups, we matched the NTG and HTG groups based on the propensity scores using global mean deviation (MD), axial length, and spherical equivalent as the matching parameters. Of the 104 young patients with OAG who met the inclusion criteria, 37 pairs of patients with NTG and HTG were selected, and the standardized mean difference after propensity score matching was 0.021. Table 1 shows the clinical and demographic characteristics of the three groups. The untreated IOP was significantly higher in the HTG group than in the NTG group, but the patients’ age, axial length, spherical equivalent, and presence of choroidal MvD did not differ between the groups. In addition, the MD value showed no intergroup difference (NTG, −2.52 ± 3.47 dB; HTG, −2.47 ± 3.14 dB; *P* = 0.897). The optic disc characteristics did not show a significant difference in the optic disc tilt ratio and the degree or direction of optic disc rotation.

**Table 1:** Clinical and Demographic Characteristics of Subgroups (mean ± SD).

	Control (n = 30)	NTG (n = 37)	HTG (n = 37)	*P* value*	*P* value^†a^	*P* value^†b^	*P* value^†c^
Age at diagnosis (years)	n/a	33.16 ± 6.16	31.92 ± 6.82	0.576^†^			
Age (years)	34.43 ± 7.44	37.35 ± 7.69	38.03 ± 8.20	0.102	0.146	0.034	0.512
Female/Male ratio	8/22	21/16	10/27	0.011^‡^	0.025^¶^	1.000^¶^	0.018^¶^
Untreated IOP (mmHg)	n/a	16.8 ± 2.2	24.2 ± 4.1	<0.001^†^			
IOP^§^ (mmHg)	15.8 ± 2.3	14.6 ± 2.2	16.0 ± 2.8	0.038	0.025	0.834	0.031
Number of eyedrops	n/a	1.66 ± 1.03	2.22 ± 1.32	0.067^†^			
Alpha agonist		4	10	0.118^‡^			
Beta-blocker		19	24				
CAI		12	19				
Prostaglandin		24	27				
Axial length (mm)	24.81 ± 1.11	25.23 ± 1.14	25.11 ± 1.60	0.410	0.169	0.514	0.524
Range	22.8 to 26.5	22.5 to 27.2	22.8 to 28.3				
Spherical equivalent (D)	−2.63 ± 1.78	−3.37 ± 2.61	−3.40 ± 2.70	0.585	0.307	0.427	0.983
Range	−5.750 to 0.375	−8.50 to 0.625	−8.50 to 0				
Corneal thickness (μm)	546.73 ± 30.24	516.94 ± 35.90	558.95 ± 33.44	<0.001	0.002	0.061	<0.001
VCDR	0.53 ± 0.07	0.78 ± 0.12	0.81 ± 0.12	<0.001	<0.001	<0.001	0.485
MD (dB)	−0.68 ± 1.23	−2.52 ± 3.47	−2.47 ± 3.14	0.070	0.043	0.029	0.897
PSD (dB)	1.66 ± 1.04	4.18 ± 4.03	3.64 ± 2.98	<0.001	<0.001	<0.001	0.627
VFI (%)	99.17 ± 1.74	93.81 ± 8.57	94.22 ± 7.73	<0.001	<0.001	<0.001	0.701
Tilt ratio	1.13 ± 0.11	1.19 ± 0.18	1.16 ± 0.12	0.095	0.078	0.046	0.685
Optic disc rotation degree	−4.22 ± 13.44	−3.56 ± 13.63	−6.60 ± 11.99	0.650	0.960	0.420	0.433
Optic disc rotation direction				0.252^‡^	1.000^¶^	0.211^¶^	0.236^¶^
Inferior	15 (50%)	18 (48.6%)	12 (32.4%)				
Superior	15 (50%)	19 (51.4%)	25 (67.6%)				
Presence of MvD	0 (0%)	7 (18.9%)	6 (16.2%)	0.046^‡^	0.014^¶^	0.029^¶^	1.000^¶^

CAI = carbonic anhydrase inhibitor; VCDR = vertical cup-to-disc ratio; HTG = high-tension glaucoma; IOP = intraocular pressure; MD = mean deviation; MvD = microvascular dropout; n/a = not applicable; NTG = normal-tension glaucoma; PSD = pattern standard deviation; SD = standard deviation; VFI = visual field index.

*Kruskal Wallis test, ^†^Mann-Whitney U test, ^‡^X^2^ test, ^¶^Fisher exact test, ^§^IOP at visit of optical coherence tomography imaging.

^a^Control vs. NTG; ^b^Control vs. HTG; ^c^NTG vs. HTG.

The VF sensitivities, retinal nerve fiber layer (RNFL) thickness, and peripapillary choroidal thickness (PCT) showed significant differences among the three groups (Table 2). Considering that the axial length influences the thickness of both RNFL and choroid, we compared the RNFL and choroid thicknesses after adjusting for axial length. Despite the absence of differences in VF sensitivities between the NTG and HTG groups in all sectors, the HTG group showed a significantly lower inferonasal RNFL thickness than the NTG group. Comparison of the PCT after adjusting for axial length indicated no intergroup difference in the average and all sectoral PCTs among the groups.

**Table 2:** Comparison of Visual Field Sensitivity, Retinal Nerve Fiber Layer Thickness, and Peripapillary Choroidal Thickness (mean ± SD).

	Control (n = 30)	NTG (n = 37)	HTG (n = 37)	*P* value^†^	*P* value^a^	*P* value^b^	*P* value^c^
Visual field sensitivity*							
Inferonasal sector	−11.20 ± 8.97	−35.49 ± 55.63	−29.43 ± 33.61	0.028	0.324^¶^	0.003^¶^	0.270^¶^
Inferotemporal sector	−22.63 ± 10.41	−78.70 ± 101.66	−67.11 ± 66.67	<0.001	<0.001^¶^	<0.001^¶^	0.581^¶^
Temporal sector	−11.83 ± 9.41	−12.05 ± 6.87	−12.08 ± 10.41	0.629	0.672^¶^	0.627^¶^	0.338^¶^
Superotemporal sector	−18.13 ± 6.96	−27.38 ± 20.08	−21.35 ± 13.50	0.073	0.030^¶^	0.480^¶^	0.107^¶^
Superonasal sector	−13.20 ± 9.77	−29.35 ± 27.60	−24.08 ± 21.78	0.002	<0.001^¶^	0.015^¶^	0.307^¶^
Nasal sector	−5.80 ± 4.33	−5.59 ± 4.65	−5.03 ± 5.97	0.407	0.960^¶^	0.250^¶^	0.251^¶^
RNFL thickness							
Average	101.07 ± 7.49	82.89 ± 16.54	80.95 ± 16.01	<0.001^‡^	<0.001^§^	<0.001^§^	1.000^§^
Inferonasal sector	110.10 ± 21.23	98.19 ± 30.52	80.73 ± 24.97	<0.001^‡^	0.262^§^	<0.001^§^	0.003^§^
Inferotemporal sector	153.27 ± 28.56	105.05 ± 47.94	97.81 ± 38.91	<0.001^‡^	<0.001^§^	<0.001^§^	0.968^§^
Temporal sector	81.27 ± 10.64	69.41 ± 14.22	70.38 ± 16.35	0.001^‡^	0.002^§^	0.006^§^	1.000^§^
Superotemporal sector	141.87 ± 15.95	108.59 ± 27.91	107.76 ± 29.19	<0.001^‡^	<0.001^§^	<0.001^§^	1.000^§^
Superonasal sector	115.60 ± 20.11	97.59 ± 24.81	94.14 ± 20.63	0.001^‡^	0.003^§^	<0.001^§^	1.000^§^
Nasal sector	73.63 ± 12.87	63.14 ± 13.95	69.14 ± 15.72	0.028^‡^	0.029^§^	1.000^§^	0.212^§^
Peripapillary choroidal thickness							
Average	139.47 ± 59.24	138.57 ± 56.75	161.79 ± 64.54	0.113^‡^	1.000^§^	0.163^§^	0.328^§^
Inferonasal sector	114.32 ± 46.91	119.03 ± 53.08	137.81 ± 58.45	0.098^‡^	1.000^§^	0.113^§^	0.443^§^
Inferotemporal sector	116.93 ± 53.62	114.38 ± 59.25	141.68 ± 71.90	0.086^‡^	1.000^§^	0.149^§^	0.208^§^
Temporal sector	145.79 ± 70.11	129.59 ± 62.65	158.16 ± 71.33	0.200^‡^	1.000^§^	0.778^§^	0.239^§^
Superotemporal sector	155.32 ± 64.12	154.41 ± 69.32	177.32 ± 81.52	0.244^‡^	1.000^§^	0.362^§^	0.603^§^
Superonasal sector	152.00 ± 67.56	162.51 ± 63.67	177.89 ± 74.62	0.151^‡^	0.756^§^	0.157^§^	1.000^§^
Nasal sector	141.46 ± 59.38	148.05 ± 59.62	169.14 ± 61.15	0.063^‡^	0.967^§^	0.063^§^	0.435^§^

HTG = high-tension glaucoma; NTG = normal-tension glaucoma; SD = standard deviation.

*Pattern deviation values for 52 visual-test points were allocated to the corresponding sector per Garway-Heath distribution map.

^†^Kruskal Wallis test, ^‡^Adjusted for difference in the axial length using ANCOVA, ^¶^Mann-Whitney U test, ^§^Post-hoc testing using Bonferroni test with adjustment for difference in the axial length.

^a^Control vs. NTG; ^b^Control vs. HTG; ^c^NTG vs. HTG.

Regarding the comparison of the peripapillary vessel density, the average vessel densities and the inferonasal sector vessel densities showed significant differences among the groups after adjusting for axial length (Table 3). Compared with normal subjects, the HTG group showed significantly reduced peripapillary vessel density globally (*P* = 0.013) by post-hoc Bonferroni test with adjustment for differences in the axial length. In addition, the reduced vessel density in the inferonasal and superotemporal sectors of the HTG group showed borderline significance. The reduction percentage of the peripapillary vessel density in the HTG group compared with that of the control group was 6.35%, 10.20%, and 12.17% in the average, inferonasal, and superotemporal sectors, respectively. However, there was no significant difference between the NTG group and normal subjects (*P* > 0.05). The vessel density in the inferonasal sector was significantly lower in the HTG group than that in the NTG group (HTG, 18.85 ± 4.19%; NTG, 21.56 ± 4.23%, *P* = 0.019).

**Table 3:** Comparison of Peripapillary Vessel Density (mean ± SD).

	Control (n = 30)	NTG (n = 37)	HTG (n = 37)	*P* value*	*P* value^†^	*P* value^‡a^	*P* value^‡b^	*P* value^‡c^
Average	24.74 ± 1.88	23.98 ± 2.30	23.18 ± 2.06	0.012	0.016	0.505	0.013	0.319
Inferonasal sector	20.98 ± 3.85	21.56 ± 4.23	18.85 ± 4.19	0.014	0.013	1.000	0.071	0.019
Inferotemporal sector	21.11 ± 4.65	18.93 ± 5.33	18.50 ± 4.96	0.088	0.093	0.264	0.113	1.000
Temporal sector	31.24 ± 3.21	29.61 ± 3.94	29.35 ± 4.17	0.107	0.120	0.305	0.152	1.000
Superotemporal sector	22.35 ± 4.18	20.80 ± 4.55	19.64 ± 3.68	0.032	0.057	0.629	0.051	0.656
Superonasal sector	22.42 ± 5.33	21.58 ± 4.74	20.12 ± 3.85	0.123	0.183	1.000	0.252	0.521
Nasal sector	24.31 ± 4.20	24.86 ± 3.96	24.80 ± 3.63	0.830	0.862	1.000	1.000	1.000

HTG = high-tension glaucoma; NTG = normal-tension glaucoma.

*ANOVA. ^†^Adjusted for difference in the axial length using ANCOVA, ^‡^Post-hoc testing using Bonferroni test with adjustment for difference in the axial length.

^a^Control vs. NTG; ^b^Control vs. HTG; ^c^NTG vs. HTG.

**Figure 1:**
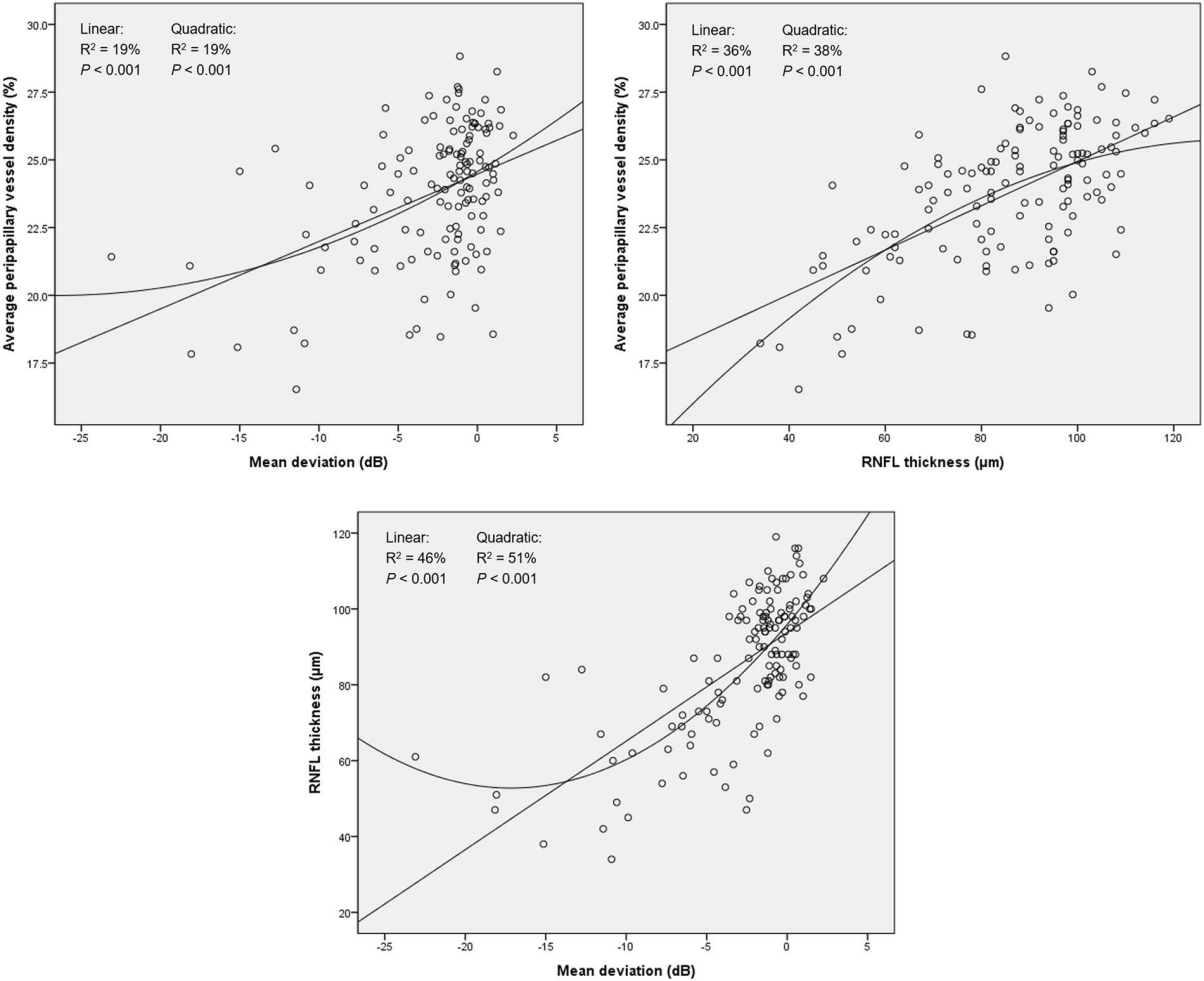
Scatter plots demonstrating linear and quadratic correlation between the average peripapillary vessel density, visual field mean deviation and average retinal nerve fiber layer (RNFL) thickness.

We evaluated the association between peripapillary vessel density and VF sensitivity, RNFL thickness, and other ocular variables in all 134 subjects via univariate and multivariate linear regression analyses (Tables 4 and 5). The following variables each showed a significant relationship with peripapillary vessel density: patient age, untreated IOP, IOP measured at the visit for optical coherence tomography (OCT) imaging, axial length, VF sensitivity, RNFL thickness, PCT, and the degree of optic disc rotation showed significant relationship with the peripapillary vessel density. Therefore, multivariate linear regression analysis was used to examine the relationship between two variables, adjusting for other confounders. As shown in Table 5, the untreated IOP showed a negative correlation with the peripapillary vessel density in the average, inferonasal and inferotemporal sectors. In addition, the average, temporal and superotemporal RNFL thicknesses showed a positive correlation with the corresponding peripapillary vessel densities. Linear and quadratic relationships between the average peripapillary vessel density with the visual field MD and the average RNFL thickness are illustrated in Fig. 1.

**Table 4:** Univariate Regression Analysis of Peripapillary Vessel Density and Visual Field Sensitivity, Structural, and Ocular Variables (n = 134).

	Peripapillary vessel density [B, *P* value, (95% CI)]						
	Average	Inferonasal	Inferotemporal	Temporal	Superotemporal	Superonasal	Nasal
Age	−0.01, 0.707, (−0.007 to 0.05)	−0.001, 0.978, (−0.11 to 0.10)	−0.11, 0.073, (−0.24 to 0.01)	−0.09, 0.063, (−0.18 to 0.01)	0.09, 0.089, (−0.01 to 0.20)	0.00, 0.997, (−0.12 to 0.12)	0.05, 0.238, (−0.04 to 0.15)
Untreated IOP	−0.18, <0.001, (−0.25 to −0.12)	−0.18, 0.005, (−0.31 to −0.06)	−0.41, <0.001, (−0.55 to −0.27)	−0.16, 0.004, (−0.28 to −0.05)	−0.18, 0.007, (−0.31 to −0.05)	−0.13, 0.065, (−0.27 to 0.01)	−0.12, 0.030, (−0.23 to −0.01)
IOP*	0.02, 0.843, (−0.14 to 0.17)	−0.34, 0.016, (−0.62 to −0.07)	0.11, 0.505, (−0.22 to 0.45)	0.12, 0.364, (−0.14 to 0.37)	−0.004, 0.978, (−0.29 to 0.28)	0.08, 0.613, (−0.23 to 0.39)	0.02, 0.846, (−0.22 to 0.27)
Axial length	0.17, 0.307, (−0.16 to 0.51)	0.74, 0.014, (0.15 to 1.33)	0.06, 0.865, (−0.66 to 0.78)	−0.27, 0.323, (−0.80 to 0.27)	−0.09, 0.767, (−0.71 to 0.52)	0.25, 0.454, (−0.41 to 0.91)	0.49, 0.065, (−0.03 to 1.00)
Visual field sensitivity^†^	0.25, <0.001, (0.16 to 0.34)	0.004, 0.648, (−0.01 to 0.02)	0.03, <0.001, (0.02 to 0.04)	0.06, 0.005, (0.02 to 0.10)	0.03, 0.007, (0.01 to 0.05)	0.02, 0.090, (−0.003 to 0.04)	0.02, 0.730, (−0.09 to 0.13)
RNFL thickness^‡^	0.08, <0.001, (0.06 to 0.10)	0.02, 0.136, (−0.01 to 0.05)	0.06, <0.001, (0.04 to 0.07)	0.11, <0.001, (0.07 to 0.14)	0.05, <0.001, (0.02 to 0.07)	0.02, 0.143, (−0.01 to 0.05)	0.04, 0.059, (−0.002 to 0.08)
PCT^¶^	−0.004, 0.275, (−0.01 to 0.003)	−0.02, 0.008, (−0.03 to −0.01)	0.01, 0.126, (−0.003 to 0.03)	0.001, 0.924, (−0.01 to 0.01)	−0.01, 0.059, (−0.02 to 0.00)	0.001, 0.897, (−0.01 to 0.01)	−0.004, 0.513, (−0.02 to 0.01)
Tilt ratio	−0.57, 0.709, (−3.56 to 2.43)	0.34, 0.900, (−5.01 to 5.70)	−1.40, 0.668, (−7.83 to 5.03)	−1.97, 0.413, (−6.71 to 2.77)	−2.40, 0.389, (−7.90 to 3.09)	0.66, 0.824, (−5.23 to 6.55)	1.10, 0.639, (−3.53 to 5.74)
Optic disc rotation degree	−0.02, 0.355, (−0.05 to 0.02)	0.03, 0.346, (−0.03 to 0.09)	−0.08, 0.042, (−0.15 to −0.003)	0.01, 0.866, (−0.05 to 0.06)	−0.03, 0.390, (−0.09 to 0.04)	−0.01, 0.680, (−0.08 to 0.05)	0.03, 0.351, (−0.08 to 0.03)

B = regression coefficient; CI = confidence interval; IOP = intraocular pressure; PCT = peripapillary choroidal thickness; RNFL = retinal nerve fiber layer.

^*^IOP at visit of optical coherence tomography imaging; ^†^Peripapillary vessel density at average and each sector regressed with mean deviation and corresponding sectoral visual field sensitivity, respectively; ^‡^Peripapillary vessel density at average and each sector regressed with average and corresponding sectoral RNFL thickness, respectively; ^¶^Peripapillary vessel density at average and each sector regressed with average and corresponding sectoral PCTs, respectively.

**Table 5:** Multivariate Regression Analysis of Peripapillary Vessel Density and Visual Field Sensitivity, Structural, and Ocular Variables (n = 134).

	Peripapillary vessel density [B, *P* value, (95% CI)]						
	Average	Inferonasal	Inferotemporal	Temporal	Superotemporal	Superonasal	Nasal
Age	n/a	n/a	−0.04, 0.457, (−0.15 to 0.07)	−0.04, 0.394, (−0.12 to 0.05)	0.09, 0.091, (−0.01 to 0.19)	n/a	n/a
Untreated IOP	−0.08, 0.016, (−0.15 to −0.02)	−0.14, 0.029, (−0.26 to −0.02)	−0.27, <0.001, (−0.41 to −0.14)	0.05, 0.367, (−0.16 to 0.06)	−0.07, 0.330, (−0.21 to 0.07)	−0.11, 0.150, (−0.25 to 0.04)	−0.09, 0.130, (−0.20 to 0.03)
IOP*	n/a	−0.20, 0.163, (−0.48 to 0.08)	n/a	n/a	n/a	n/a	n/a
Axial length	n/a	0.61, 0.042, (0.02 to 1.20)	n/a	n/a	n/a	n/a	0.69, 0.013, (0.15 to 1.24)
Visual field sensitivity^†^	n/a	n/a	0.02, 0.005, (0.01 to 0.03)	0.02, 0.254, (−0.02 to 0.06)	0.01, 0.550, (−0.02 to 0.03)	0.02, 0.215, (−0.01 to 0.04)	n/a
RNFL thickness^‡^	0.07, <0.001, (0.05 to 0.09)	n/a	0.02, 0.065, (−0.001 to 0.04)	0.09, <0.001, (0.05 to 0.13)	0.04, 0.010, (0.01 to 0.06)	n/a	0.06, 0.021, (0.01 to 0.10)
PCT^¶^	n/a	−0.01, 0.084, (−0.03 to 0.002)	n/a	n/a	−0.01, 0.192, (−0.02 to 0.004)	n/a	n/a
Optic disc rotation degree	n/a	n/a	−0.05, 0.139, (−0.10 to 0.02)	n/a	n/a	n/a	n/a

B = regression coefficient; CI = confidence interval; IOP = intraocular pressure; n/a = not applicable; PCT = peripapillary choroidal thickness; RNFL = retinal nerve fiber layer.

^*^IOP at visit of optical coherence tomography imaging; ^†^Peripapillary vessel density at average and each sector regressed with mean deviation and corresponding sectoral visual field sensitivity, respectively; ^‡^Peripapillary vessel density at average and each sector regressed with average and corresponding sectoral RNFL thickness, respectively; ^¶^Peripapillary vessel density at average and each sector regressed with average and corresponding sectoral PCTs, respectively.

## Discussion

In the present cross-sectional study including young patients with OAG and age-matched normal subjects, the peripapillary vessel density was significantly reduced in the HTG group compared with the normal control group, with absence of difference between the NTG and the normal control groups. A comparison of peripapillary vessel density between the axial length, refractive error, and MD-matched NTG and HTG groups showed that the vessel density at the inferonasal sector alone was significantly lower in the HTG group. Likewise, the peripapillary RNFL thickness showed a sectoral difference between the HTG and NTG groups. In contrast, the peripapillary vessel density did not differ significantly between the normal and the NTG eyes. Furthermore, we demonstrated a negative correlation between untreated IOP and peripapillary vessel density. To our knowledge, this is the first study to investigate the peripapillary vessel density in young OAG patients with respect to the initial untreated IOP level.

Recent advances in OCTA have enabled noninvasive *in-vivo* visualization of the vasculature around the optic disc. Previous studies reported peripapillary microvascular attenuation in patients with glaucoma compared with normal subjects^18–21^. Liu and colleagues first reported the presence of reduced peripapillary vessel density in glaucomatous eyes compared with normal eyes^19^. Since then, several studies have demonstrated spatial concordance between the RNFL defect and reduced peripapillary vessel density, as well as its diagnostic role in patients with glaucoma^21,22,26,27^. Reports indicated a decrease in peripapillary vessel density at a location corresponding to the VF defect in glaucomatous eyes^28,29^. However, few studies have compared the peripapillary microvasculature between NTG and HTG groups using OCTA^24,30–32^.

Scripsema and colleagues reported a decrease in the peripapillary capillary density of patients with NTG and HTG compared with that of normal subjects, with reduced peripapillary vessel density in patients with HTG versus NTG^31^. In contrast, a recent study reported a significant reduction in the perfused peripapillary vessel density in patients of HTG and NTG groups than in age- and sex-matched normal subjects, with a more prominent reduction in eyes with NTG^24^. However, Bojikian and colleagues demonstrated the absence of significant differences in blood flux within the prelaminar cribrosa between the HTG and NTG groups^30^. In the present study, a significantly lower vessel density was found in the inferonasal sector alone in the HTG compared with the NTG group. Furthermore, the peripapillary vessel density was reduced significantly in the HTG eyes than in the normal eyes, with no difference in peripapillary vessel density between the NTG and normal control groups. The reasons for the inconsistent results between the current and previous studies are unclear; however, such inconsistencies may be attributed to differences in ethnicity, age, OCT devices used, and glaucoma severity of the respective study participants, as well as the inherent limitation of the propensity score matching used for matching two groups. In our study, all the study participants were Korean, whereas previous studies included Hispanic or Chinese patients. Moreover, the subjects’ ages in the previous studies ranged from 50.9 to 67.9 years, which was far higher than that of our cohort (mean: 37.7 years). In addition, our study included a greater number of patients with milder glaucomatous damage (mean MD: NTG, −2.52 dB; HTG, −2.47 dB) than that in previous studies (mean MD: −4.79 to −9.76 dB). The inclusion of young patients in our study may have minimized the possible confounding effect of coexisting systemic diseases on the ocular blood flow or the parapapillary retinal microvasculature.

In the present study, the inferonasal sector was the only sector demonstrating significant differences in both peripapillary vessel density and RNFL thickness between the HTG and NTG groups, which is in accordance with previous findings of lower RNFL thickness related to the lower vessel density^21,22,26,27^. However, although the RNFL thickness was significantly lower in the NTG group than that in the normal subjects, the peripapillary vessel density showed no intergroup difference. Presently, we have no clear explanation for this finding; however, it is likely that the influencing factors are as follows. First, the current study included a higher number of patients with early glaucomatous damage. Changes in RNFL thickness may precede or not coincide with those involving the peripapillary microvasculature in early stages of glaucomatous damage. Consistent with our finding, Kim *et al*.^33^ reported that eyes diagnosed with preperimetric glaucoma and normal eyes showed a similar peripapillary microvascular density in the area of RNFL defect, whereas eyes with perimetric glaucoma showed a significant decrease in vessel density in the area of RNFL thinning. Second, the different levels of untreated baseline IOP may have contributed to differences in the pathogenetic mechanisms of glaucoma in young patients. In the present study, the young OAG patients showed moderate myopia. Both the optic disc tilt and rotation are characteristic morphologic changes of a myopic optic disc and considered to be the consequence of progressive posterior scleral remodeling during axial elongation^34,35^. As the axial elongation progresses slowly with the morphologic changes of the optic disc, the retinal vascular system may have adapted to the microvasculature in the peripapillary region, exerting different regulatory capability of the vasculature to morphologic changes of the optic disc in young glaucoma patients. The autoregulatory system of the retina has the capability of blood-flow regulation under various conditions^36,37^. In addition to the morphologic changes shown in the eyes with NTG, the elevated IOP may induce additional direct mechanical stress on the retinal vasculature of the eyes with HTG, thereby increasing the magnitude and rate of decrease in peripapillary vessel density. Furthermore, the results of the multivariate linear regression model revealed a negative correlation of untreated IOP with peripapillary vessel density. Third, differences in the usage of glaucoma medication may have contributed to such differences between the two groups. Compared with NTG patients, the HTG patients used glaucoma medications more frequently with borderline significance, including additional topical beta-blockers, which may have affected the ocular perfusion pressure^28^.

Several studies using OCTA have identified localized MvD in the peripapillary choroid in patients with glaucoma^23,28^. Sung and colleagues demonstrated positive relationships of the ovality index and the degree of inferior optic disc rotation with the increased risk of deep peripapillary microvascular attenuation in young myopic subjects, indicating a possible association between abnormal choroidal circulation and deformation of optic nerve head (ONH)^38^. In this study, we investigated the presence of peripapillary choroidal MvD to identify the differences in abnormal local choroidal circulation between the NTG and HTG groups. However, the detection rate of MvD was similar between the two groups. The mechanism underlying this finding is not clearly understood. However, differences in the origin of blood supply may contribute to differences in vascular changes that occur in the peripapillary retinal microvasculature and choroidal regions of eyes with HTG and NTG. In addition, different regulation of choroidal and ONH blood flow during changes in ocular perfusion pressure may play a role in this observation^39^.

This study has some limitations. First, its retrospective design and small sample size may limit generalization of the present findings. Moreover, most of the included patients showed relatively early glaucoma. Second, we did not measure the blood pressure and full diurnal curves of IOP. However, we obtained the untreated IOP readings at ≥2 time-points; moreover, all patients with NTG underwent brain imaging to rule out the presence of any associated neurological lesion. Third, since the study measurements involved a single Korean ethnic group with a higher NTG prevalence than HTG, the study results may be different in other populations with a higher prevalence of HTG. Further studies including more subjects of different ethnic backgrounds may yield additional information on the peripapillary vessel density in young patients with OAG. Finally, because this study is a cross-sectional study, the causal relationship between glaucomatous damage and reduction in peripapillary vessel density has not been identified. Previously, Lee and colleagues reported that attenuation of retinal microvasculature was associated with secondary loss of capillaries in the region of glaucomatous RNFL atrophy^40^. In contrast, other studies reported that reduced peripapillary vessel density can occur before structural damage, suggesting that attenuation of retinal microvasculature may cause the glaucomatous damage^41,42^. A longitudinal study is warranted to clarify the issue. Nevertheless, the strength of our study is that the confounding effect of systemic disease or cataract, which can affect the measurement of peripapillary vessel density, was minimized by excluding patients over 40 years of age. In addition, there were no intergroup differences in VF sensitivity at all sectors by matching the MD value of patients in each group. Furthermore, we selectively measured the peripapillary capillary density by excluding signals of large retinal vessels. Recently, Holló reported that subtracting signals of large retinal vessels from the peripapillary vessel density enhanced the accuracy of evaluation of glaucoma progression^43^.

In conclusion, despite the similarity between sectoral VF sensitivities in young patients with NTG and HTG, eyes with elevated IOP showed a reduction in peripapillary vessel density and RNFL thickness in the inferonasal sector. In addition, the peripapillary vessel density was decreased in eyes with HTG compared to normal subjects, whereas the peripapillary vessel density was similar between eyes with NTG and normal eyes. These results suggest that different initial untreated IOP levels may affect the peripapillary vessel density differently in young patients with OAG. However, additional research is needed to evaluate the changes of peripapillary vessel density in the eyes with more advanced stage of the disease.

## Methods

We reviewed the medical records of young patients with OAG who visited Korea University Anam Hospital from March 2016 to March 2018. The Institutional Review Board of the Korea University Anam Hospital approved the study and the need for written informed consent was waived by our Review Board. This retrospective study was performed according to the tenets of the Declaration of Helsinki.

In this study, the inclusion criteria of the young patients with OAG were: (1) age 40 years or younger at the time of diagnosis; (2) spherical equivalent ≥−9 or ≤+3 diopters; (3) presence of open anterior chamber angle based on static gonioscopy; and (4) presence of glaucomatous ONH changes and corresponding VF defects. Exclusion criteria were any of the following: (1) presence or history of secondary OAG (inflammatory, neovascular, pigmentary, pseudoexfoliation, post-traumatic, or steroid-induced glaucoma); (2) history of intraocular or refractive surgery; (3) medical diseases such as systemic hypertension, diabetes mellitus, heart disease, or autonomic dysregulation; and (4) presence or history of topical or systemic steroid use. The patients were divided into two groups according to the level of untreated IOP measured by Goldmann applanation tonometry. The untreated IOP was measured on at least two different time points before the use of glaucoma medication. The HTG group showed IOP ≥ 22 mmHg and the NTG group, IOP ≤ 19 mmHg. If both eyes were eligible for inclusion in the study, we selected the eye with the lower MD value from each patient for analysis. The NTG and HTG groups were matched based on the propensity scores using global MD, axial length, and spherical equivalent as the matching parameters to compare the peripapillary vessel density.

The control participants showed no evidence of retinal pathology or glaucoma, no family history of glaucoma, IOP ≤ 21 mmHg without any history of elevated IOP, open angle based on static gonioscopy, normal-appearing optic discs, global circumpapillary RNFL thickness measured on OCT within the 95% confidence interval (CI) of the mean, and normal VF tests defined as pattern standard deviation (PSD) within the 95% confidence limits and a Glaucoma Hemifield Test within normal limits.

All patients received an ocular examination, including best corrected visual acuity measurement, refraction test, slit-lamp examination, IOP measurement with Goldmann applanation tonometry, central corneal thickness measurement using specular microscopy (SP-2000P, Topcon, Tokyo, Japan), axial length measurement using IOLMaster (Carl Zeiss Meditec, Jena, Germany), dilated 30-degree stereoscopic fundus photography and 50-degree red-free photography using a FF 450 plus IR camera (Carl Zeiss Meditec Inc., Dublin, CA), Humphrey 24-2 Swedish Interactive Thresholding Algorithm standard automated perimetry (Carl Zeiss Meditec, Dublin, CA, USA), and swept-source OCT and OCTA (DRI OCT Triton, Topcon, Tokyo, Japan).

### Optical coherence tomography angiography

The peripapillary area was imaged using a swept-source OCT device (DRI OCT Triton, Topcon) which provides a central wavelength of 1050 nm and acquisition speed of 100,000 A-scans per second. Images were acquired from 4.5 × 4.5-mm cube scans and each cube was consisted of 320 clusters of four repeated B-scans centered on the optic disc. The pupil was fully dilated with 0.5% tropicamide and 0.5% phenylephrine (Mydrin-P, Santen, Osaka, Japan) before the examination and the subjects were directed to focus their gaze on the internal fixation target to optimize the image quality. Patients whose images had poor-quality were excluded using the following criteria: (1) inadequate signal strength <50; (2) motion artifacts; (3) inadequate focus or poor clarity; or (4) segmentation failure.

**Figure 2:**
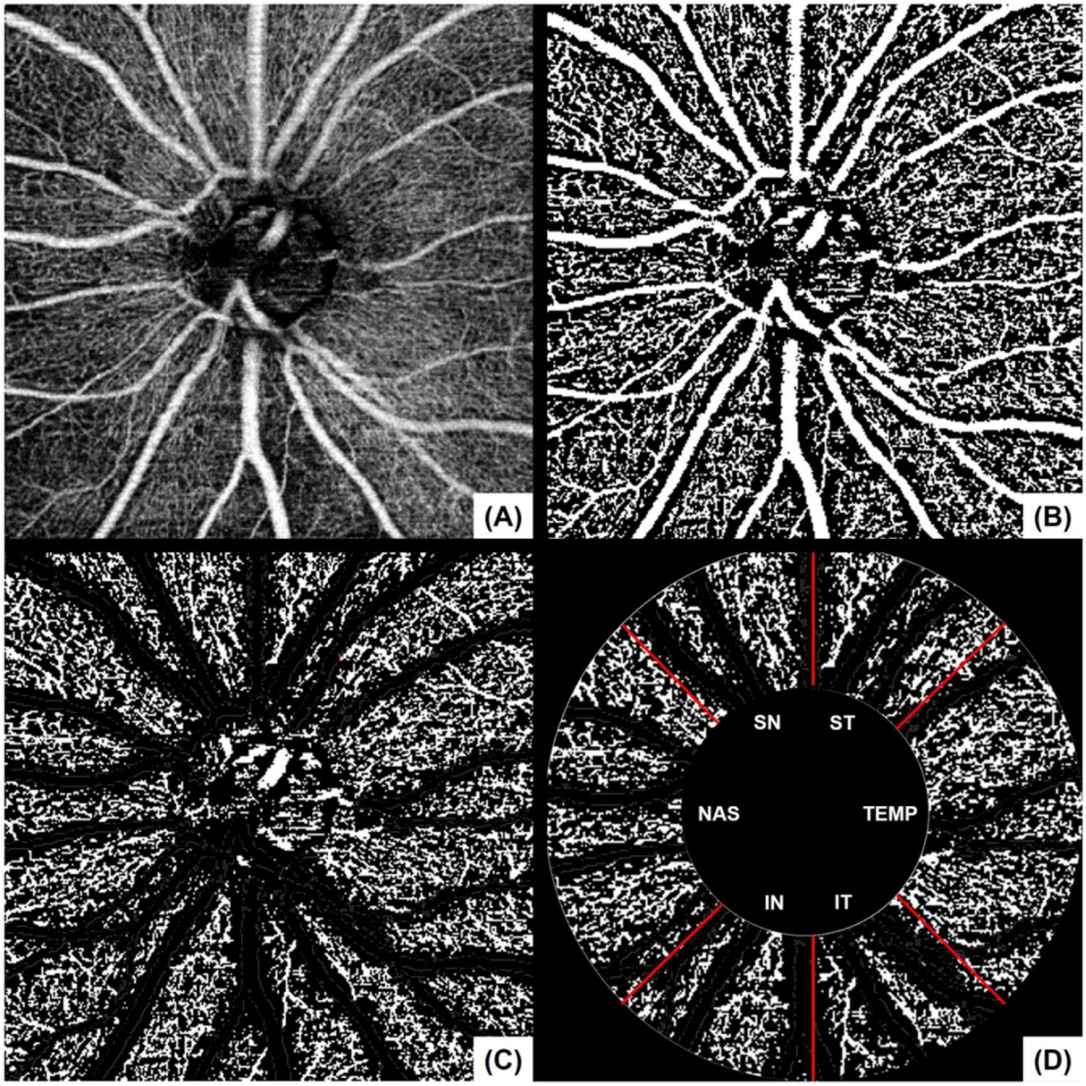
Measurement of the peripapillary vessel density of the left eye. En face image of the radial peripapillary capillary (**A**) was processed to delineate the retinal vasculature from background noise (**B**). After image processing, the major vessels were removed (**C**), followed by division of the peripapillary region into six sectors to measure the vessel density (**D**). The peripapillary vessel density was calculated as the percentage of the sampled area comprising vessels detected based on the thresholding parameters.

The en face image of the superficial retinal slab was generated based on the automated layer segmentation performed by the OCT instrument software. The inner boundary of the superficial retinal slab was 2.5 µm beneath the internal limiting membrane, and the outer boundary was 15.6 µm beneath the interface of the inner plexiform layer/inner nuclear layer. To delineate the retinal vasculature from background noise, the Auto Local Threshold plug-in of ImageJ (version 1.52, National Institutes of Health, Bethesda, MD, USA) was used to process the en face images^44^. Local thresholding was performed using the “mean method” with a radius of 10 and a C value of −10^44^. After processing the en face image, we removed the major vessels using Photoshop software (version 11.0, Adobe, San Jose, CA, USA). To measure the vessel density, we defined the peripapillary region as the 750-μm-wide elliptical annulus extending from the optic disc boundary and divided the region into six sectors (superotemporal, superonasal, nasal, inferonasal, inferotemporal, and temporal; used in the algorithm of RNFL thickness). The peripapillary vessel density was calculated as the percentage of the sampled area occupied by vessels detected by the thresholding parameters (Fig. 2).

The en face image of the deep layer, extending from the retinal pigment epithelium up to 390 μm below the Bruch’s membrane, was generated through the OCT instruments’ software and we evaluated the choroidal microvasculature in the peripapillary area. Microvasculature dropout was defined as a focal sectoral capillary dropout with no visible microvascular network identified in the deep layer en face images^23^.

### Thickness of RNFL and peripapillary choroid

To investigate the RNFL thickness and peripapillary choroidal thickness, we used the standard protocol of 360°, 3.4-mm diameter scan around the optic disc. The circle scan produced 12 sectors around the disc numbered from 1 o’clock to 12 o’clock position; numbering was clockwise in the right eye and counterclockwise in the left eye. The RNFL thickness and PCT were automatically derived from a scan using built-in automated segmentation software. Retinal nerve fiber layer thickness and PCT were also divided into six sectors, and the values were used for the analysis.

### Evaluation of optic disc appearance

The optic discs were independently evaluated in a random order and in a blinded fashion by two glaucoma specialists (CY and JHP) using ImageJ version 1.52 software. Optic disc tilt was identified based on the tilt ratio, which is the ratio of the longest and shortest diameters of the optic disc. Optic disc rotation was defined as the deviation of the long axis of the optic disc from the vertical meridian, identified as a vertical line 90 degrees from a reference line connecting the center of the disc to the center of the macula. Superior rotation represented clockwise rotation of the optic disc in the right eye, or counter-clockwise rotation of the optic disc in the left eye; inferior rotation referred to the counter-clockwise rotation of the optic disc in the right eye, or clockwise rotation of the optic disc in the left eye. Positive and negative values indicated inferior and superior rotation, respectively^35^.

### Visual field defect locations

All subjects underwent VF testing using the 24-2 pattern Swedish Interactive Threshold Algorithm on the Humphrey Field Analyzer within 6 months of OCT/OCTA imaging. All eyes included in the VF analysis had at least two reliable tests and consistent VF defects corresponding to the optic disc changes. VF tests with a fixation loss rate ≤20% and false-negative and false-positive rates ≤20% were determined as reliable VF tests. To evaluate the sectoral structure-function relationships, we allocated the pattern deviation values of 52 VF points to the corresponding sector according to Garway-Heath distribution map^45^.

### Statistical analysis

Normality of distribution was assessed using the Shapiro-Wilk normality test, and parametric or nonparametric tests were subsequently used. Categorical data analysis was conducted using Fisher’s exact test or chi-square test. After considering the correlation between sectors, we performed analysis of covariance (ANCOVA) to compare the peripapillary vessel density among the three groups, and post-hoc Bonferroni test to assess the intergroup differences. Relationships between the peripapillary vessel density, visual field MD and RNFL thickness were evaluated by simple linear or quadratic models. To determine the clinical variables associated with peripapillary vessel density, univariate and multivariate linear regression analyses were performed. Variables with a *P* value less than 0.10 in the univariate analysis were entered into the multivariate analysis. *P* value < 0.05 indicated statistical significance. All statistical analysis was performed using SPSS software version 21.0 (IBM corp., Armonk, NY, USA).

## Supplementary information

Supplementary Table.

## Data Availability

The datasets generated during and/or analysed during the current study are available from the corresponding author on reasonable request.
