# Supplementary material for: Peripapillary Vessel Density in Young Patients with Open-Angle Glaucoma: Comparison between High-Tension and Normal-Tension Glaucoma
Source: Sci Rep. 2019 Dec 16;9:19160. doi: 10.1038/s41598-019-55707-5 (PMC6914787; doi:10.1038/s41598-019-55707-5)
Supplement: Supplementary file 1 — Supplementary Table. [file 41598_2019_55707_MOESM1_ESM.pdf]

[Supplementary Information]

Peripapillary Vessel Density in Young Patients with Open-Angle Glaucoma: Comparison between High-Tension and Normal-Tension Glaucoma

Ji-Hye Park, Chungkwon Yoo*, Yong Yeon Kim

Department of Ophthalmology, Korea University College of Medicine, Seoul, Korea

Corresponding author: Chungkwon Yoo, MD, PhD

Department of Ophthalmology, Korea University College of Medicine

126-1 Anam-dong 5-ga, Sungbuk-gu, Seoul 02841, Korea

E-mail: ckyoomd@korea.ac.kr

Tel: 82-2-920-5521

Fax: 82-2-924-6820

Supplementary Table. Clinical and Demographic Characteristics of Subgroups (mean ± SD)

	Control (n=30)	NTG (n=57)	HTG (n=47)	*P* value*	*P* value†a	*P* value†b	*P* value†c
Age at diagnosis (years)	n/a	33.32 ± 5.71	32.23 ± 6.52	0.596†			
Age (years)	34.43 ± 7.44	37.35 ± 6.66	38.30 ± 7.63	0.044	0.114	0.016	0.203
Female/Male ratio	8/22	34/23	17/30	0.005‡	0.006¶	0.459¶	0.019¶
Untreated IOP (mmHg)	n/a	16.4 ± 2.3	25.3 ± 6.2	<0.001†			
IOP§ (mmHg)	15.8 ± 2.3	14.2 ± 2.4	16.0 ± 3.0	0.001	0.003	0.829	0.002
Number of eyedrops	n/a	1.47 ± 0.98	2.30 ± 1.25	0.001†			
Alpha agonist		4	11	<0.001‡			
Beta-blocker		25	33				
CAI		14	11				
Prostaglandin		40	35				
Axial length (mm)	24.81 ± 1.11	25.39 ± 1.06	24.90 ± 1.58	0.046	0.035	0.951	0.040
Range	22.8 to 26.5	22.5 to 27.8	22.7 to 28.3				
Spherical equivalent (D)	-2.63 ± 1.78	-4.11 ± 2.72	-2.92 ± 2.65	0.021	0.014	0.942	0.026
Range	-5.750 to 0.375	-8.750 to 0.625	-8.500 to 1.000				
Corneal thickness (μm)	546.73 ± 30.24	516.60 ± 33.19	553.53 ± 34.98	<0.001	<0.001	0.202	<0.001
VCDR	0.53 ± 0.07	0.78 ± 0.13	0.82 ± 0.13	<0.001	<0.001	<0.001	0.102
MD (dB)	-0.68 ± 1.23	-2.27 ± 3.04	-4.69 ± 5.90	0.004	0.034	0.001	0.081
PSD (dB)	1.66 ± 1.04	3.80 ± 3.56	5.18 ± 1.62	<0.001	<0.001	<0.001	0.296
VFI (%)	99.17 ± 1.74	94.81 ± 7.27	88.21 ± 16.26	<0.001	<0.001	<0.001	0.217
Tilt ratio	1.13 ± 0.11	1.20 ± 0.17	1.15 ± 0.12	0.095	0.045	0.504	0.126
Optic disc rotation degree	-4.22 ± 13.44	-2.02 ± 12.75	-5.77 ± 11.81	0.354	0.728	0.552	0.126
Optic disc rotation direction				0.075‡	0.654¶	0.234¶	0.030¶
Inferior	15 (50%)	16 (34.0%)	32 (56.1%)				
Superior	15 (50%)	31 (66.0%)	25 (43.9%)				
Presence of MvD	0 (0%)	16 (28.1%)	12 (25.5%)	0.006‡	0.001¶	0.002¶	0.827¶

CAI = carbonic anhydrase inhibitor; VCDR = vertical cup-to-disc ratio; HTG = high-tension glaucoma; IOP = intraocular pressure; MD = mean deviation; MvD = microvascular dropout; n/a = not applicable; NTG = normal-tension glaucoma; PSD = pattern standard deviation; SD = standard deviation; VFI = visual field index.

*Kruskal Wallis test, †Mann-Whitney U test, ‡X^2 test, ¶Fisher exact test, §IOP at visit of optical coherence tomography imaging.

[a]Control vs. NTG; [b]Control vs. HTG; [c]NTG vs. HTG.
